# Transparent Structures for ZnO Thin Film Paper Transistors Fabricated by Pulsed Electron Beam Deposition

**DOI:** 10.3390/mi15020265

**Published:** 2024-02-12

**Authors:** Florin Gherendi, Daniela Dobrin, Magdalena Nistor

**Affiliations:** National Institute for Lasers, Plasma and Radiation Physics (INFLPR), P.O. Box MG-36, 077125 Magurele-Bucharest, Romania; florin.gherendi@inflpr.ro (F.G.); daniela.dobrin@inflpr.ro (D.D.)

**Keywords:** thin film transistor, zinc oxide, fabrication, characterization

## Abstract

Thin film transistors on paper are increasingly in demand for emerging applications, such as flexible displays and sensors for wearable and disposable devices, making paper a promising substrate for green electronics and the circular economy. ZnO self-assembled thin film transistors on a paper substrate, also using paper as a gate dielectric, were fabricated by pulsed electron beam deposition (PED) at room temperature. These self-assembled ZnO thin film transistor source–channel–drain structures were obtained in a single deposition process using 200 and 300 µm metal wires as obstacles in the path of the ablation plasma. These transistors exhibited a memory effect, with two distinct states, “on” and “off”, and with a field-effect mobility of about 25 cm^2^/Vs in both states. For the “on” state, a threshold voltage (V_th on_ = −1.75 V) and subthreshold swing (S = 1.1 V/decade) were determined, while, in the “off” state, V_th off_ = +1.8 V and S = 1.34 V/decade were obtained. A 1.6 μA maximum drain current was obtained in the “off” state, and 11.5 μA was obtained in the “on” state of the transistor. Due to ZnO’s non-toxicity, such self-assembled transistors are promising as components for flexible, disposable smart labels and other various green paper-based electronics.

## 1. Introduction

In emerging applications, such as flexible displays and sensors for wearable devices, where paper is a promising substrate for green electronics, thin film transistors (TFTs) are increasingly in demand. Cellulose, the fiber used to make paper, is one of the world’s most abundant biomaterials, with applications in medicine, food, cosmetics, electronics, and engineering [1]. Paper is an attractive substrate for flexible electronic devices because it is renewable, non-toxic, and flexible, and it has robust mechanical and dielectric properties [2]. The low-cost synthesis of eco-friendly devices on paper will help to reduce electronic waste and create a sustainable future in a circular economy, as cellulose is 100% biodegradable and is, therefore, the most recyclable of all the materials that contribute to the solid waste stream [3].

Zinc oxide (ZnO) is one of the most studied transparent n-type conducting oxides in the form of thin films or nanostructures for optoelectronic devices due to its tunable electrical and optical properties via growth parameters and doping [4,5]. The typical fabrication of ZnO thin film transistors on silicon and glass substrates includes physical vapor deposition technologies like magnetron sputtering for channel and evaporation for contact electrodes [6,7], pulsed laser deposition [8], pulsed electron beam deposition (PED) [9] and, more recently, solution-based printing and chemical methods [10]. Although ZnO requires higher temperatures to achieve good crystallinity and charge-carrier mobility, the use of paper as a substrate restricts the growth temperature of the oxide thin films to room temperature or below 150 °C.

Carcia et al. reported the first ZnO thin film transistors on Si substrates, fabricated by radio-frequency magnetron sputtering, with remarkable transistor performance, including a field-effect mobility > 2 cm^2^/V s and an on/off ratio > 10^6^ [6]. These ZnO thin films showed their potential as components for flexible electronics on temperature-sensitive substrates due to their high optical transparency (>80% at wavelength > 400 nm) and resistivity of ~10^5^ Ωcm. Fortunato et al. fabricated high-performance ZnO thin film transistors on glass substrates at room temperature [7]. With a threshold voltage of 19 V, a saturation mobility of 27 cm^2^/Vs, a gate-voltage swing of 1.39 V/decade, and an on/off ratio of 3 × 10^5^, the bottom-gate configuration ZnO thin film transistor was operating in an enhanced mode.

In spite of the promising properties of ZnO thin film transistors and the opening of the path for the rapid development of metal oxide TFTs over the past few decades, their performance has been surpassed by that of amorphous oxide semiconductors like indium−gallium−zinc oxide (IGZO) [11]. Being amorphous, they have the advantage of exhibiting improved TFT performance and reliability in comparison with amorphous Si and organic TFTs, which is attributed to their high electron mobility, superior channel material homogeneity due to their lack of grain boundaries, remarkable transparency in the visible range, and a deposition temperature at or near room temperature, resulting in good adaptability to flexible substrates [12]. However, they present some instabilities due to trap levels near the film surface, which become filled and are slowly and unevenly de-trapped [13,14]. They also include elements like indium and gallium, which are expensive and not ideal for green electronics, for which ZnO is much better adapted. The fact that ZnO thin films are readily polycrystalline with a hexagonal würtzite structure close to room temperature on various substrates gives them the disadvantage of reducing the mobility of the charge carriers (electrons) due to dislocations or impurities at the grain boundaries, hence influencing their resulting performances in transistors. Some attempts have been made to obtain amorphous zinc oxide thin films and, thus, to eliminate the grain boundaries by pulsed laser deposition and sputtering at cryogenic temperatures [15,16,17] by using chemical methods [18,19] and, recently, by enhancing the oxygen deficiency in PED [20]. However, these attempts are neither expensive in their applications (cryogenic temperatures) nor does the large oxygen deficiency benefit the fabrication of the transistor’s components, which require almost ideal ZnO stoichiometry. Rogers [21] demonstrated the successful growth of amorphous ZnO films on paper and mylar using pulsed laser deposition at room temperature, achieving relatively low resistances. This highlights the promising performance of the ablation method for such transistor films, which would eliminate the inconvenience of the post-deposition annealing step typically used in other deposition methods to improve the optoelectronic properties of the films.

The path to oxide-based electronics on paper substrates was pioneered by Fortunato et al. [22], who made the first paper transistor using cellulose paper, both as a gate dielectric and as a device substrate. A gallium−indium−zinc oxide thin film grown at room temperature via RF magnetron sputtering was used as an n-type channel semiconductor in this hybrid paper transistor. Patterned Al films with a shadow mask were deposited by evaporation as the source and drain contacts, while a conducting indium-zinc-oxide gate film was deposited as the electrode on the other side of the paper by magnetron sputtering. With a channel saturation mobility of 30 cm^2^/Vs, a threshold voltage close to zero, a gate-voltage swing of ~0.8 V/decade, and an on/off ratio of >10^4^, the paper transistor exhibited properties comparable to those fabricated on hard substrates (glass or silicon), despite the roughness of the paper surface. Improvements to the cellulose fibril’s composition, density, and functionalization have led to a smoother paper surface and, therefore, to higher quality deposited thin films on the paper, resulting in reduced operating voltages and leakage currents, thus enhancing inorganic or organic transistor performances [3,22,23,24,25,26,27,28].

Despite these results, the main challenge remains to obtain transistor components (channel and electrodes) from the same material in a single deposition process at room temperature in order to reduce manufacturing costs compared to those associated with silicon technology; therefore, the use of simple techniques is essential. Following our previous work, ZnO self-assembled thin film transistors were fabricated by the ablation method with a pulsed electron beam, also named “pulsed electron deposition” (PED), at room temperature. In previous research, our group reported on In_2_O_3_ self-assembled thin film transistors on glass and paper using an original and low-cost approach [9,29,30]. ZnO can prove itself more sustainable, cheaper, and non-toxic as a replacement for In_2_O_3_ thin films, but obtaining the thin film properties suitable for transistors—a high enough conductivity for source–drain and gate contacts and non-degenerated/low carrier density semiconductor properties for the channel—can be challenging. The experimental conditions for obtaining functional transparent structures for ZnO thin film transistors were identified and optimized by varying the PED deposition parameters. The effect on the electrical and optical properties was determined using ZnO witness (“model”) thin films. For obtaining self-assembled structures in a single deposition process, shadow masks (mechanical masks) placed at a controlled distance from the substrate were used. The thin film field-effect transistors’ source−channel−drain structures were deposited as self-assembled ZnO thin films on paper. These structures were investigated by the profilometry technique in order to determine the channel thickness. Electrical measurements of the transistors were carried out by a homemade semiconductor parameter analyzer in the air, in the dark, and at room temperature.

## 2. Materials and Methods

The PED method is an ablation deposition method, similar to pulsed laser deposition (PLD), using a pulsed electron beam instead of the nanosecond ultraviolet laser beam. The focused electron beam is produced in a channel-spark discharge attached to the deposition chamber, as described in detail previously [20]. The electron beam in PED is pulsed (about 100–110 ns at full-width at half-maximum), very intense (approximately hundreds of amperes), and has a polyenergetic energy distribution of electrons, which makes it possible to ablate, i.e., to melt, and vaporize and generate an ablation plasma from a solid target, as is done in the pulsed laser deposition process [20]. The electron beam interacts with the ZnO target at an angle of 45 degrees. An ablation plasma is formed and propagates towards the substrate located at a 4–5 cm distance. The typical fluence in our experiments varies up to 2.5 J/cm^2^, and the working gas is oxygen in the pressure range of 1–2 × 10^−2^ mbar. PED is a versatile and cost-effective thin film deposition method that enables the adjustment of deposition parameters to precisely control the composition, morphology, thickness, and physical properties of various thin films [5,9,20,29,30].

**Figure 1:**
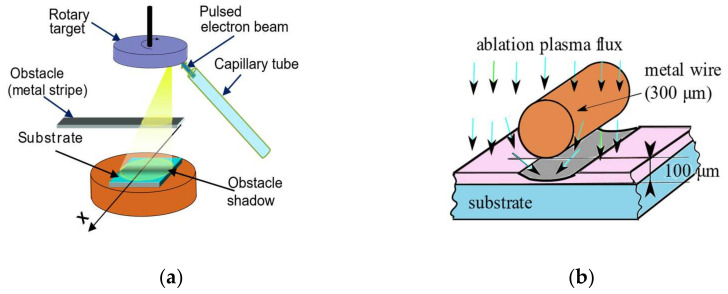
Schematic drawing of the PED deposition setup with an obstacle inserted in the ablation plasma: (**a**) PED installation with 10 mm obstacle; (**b**) deposition of a self-assembled ZnO thin film with a source−channel−drain structure using a 300 µm metal wire as an obstacle.

For the production of transistors with a minimum number of deposition steps, an original approach has been used in which a mechanical obstacle is introduced into the ablation plasma as it propagates toward the substrate. The experimental setup (Figure 1a) for the mechanical obstacle PED deposition is similar to that used in previous research of the group for In_2_O_3_ thin films [9,29,30].

The obstacle introduced in the ablation plasma consists either of a metal strip with a width of 10 mm to obtain the large structures (“model system”), as in Figure 1a, or by a reduction in scale [9] of a metal wire with a diameter between 200 and 300 μm for obtaining transistor structures (Figure 1b). The distance from the deposition substrate to the obstacle is of the same order of magnitude as the width of the obstacle. Thus, the 10 mm wide obstacle was placed 19 mm away from the substrate, and such zinc oxide films deposited on glass and paper substrate were used in the preliminary research for optimizing the working conditions in oxygen at room temperature. With this purpose, optical transmission in the ultraviolet and visible spectral ranges and Hall measurements at room temperature were performed on these “model” ZnO thin films (not shown here). To keep the proportions of the large obstacle setup, the 200 and 300 μm diameter wires were placed at a distance of 100 μm from the substrate in order to fabricate ZnO self-assembled depositions for the source−channel−drain structures of thin film transistors.

It has been shown for indium oxide thin films grown by PED that when the working gas used is oxygen, the self-assembled deposition of a semiconductor oxide thin film behind the obstacle has a lower charge-carrier density in that region compared with the non-shadowed wings [9,29,30]. For the ablation deposition method, it has been proven by isotope tracing that a small fraction of the oxygen in oxide thin films comes from the working gas, and the resulting film does not contain only oxygen from the target [31,32]. In the case of indium oxide thin films, we have shown that a reduction in the ablation plasma density behind the obstacle leads to a higher proportion of oxygen incorporated in the film grown in the shadow of the obstacle, thus obtaining a lower concentration of oxygen vacancies. As in the semiconductor oxide films, the charge-carrier density depends on the concentration of oxygen vacancies [33,34]; at lower oxygen pressures in PED, we can even obtain degenerate semiconductor oxide films [5,20,35]. By very slightly changing the working oxygen pressure in PED, we can also tune the electrical and optical properties of both In_2_O_3_ [35] and ZnO thin films [5,20] despite the differences between these two transparent conducting oxides. This is a specific feature of PED in comparison to PLD [4,8] and magnetron sputtering [6,7,36] and is related to the contribution of the high kinetic energy of ablation plasma species to the thin film growth on the substrate. On the other hand, the background gas slows down the ablated species by scattering, and there are also reactions with the background gas, resulting in a reduction in the kinetic energies of the species arriving at the substrate so that the heating of the substrate by the ablation plasma is avoided. During the deposition of the ZnO film on the paper substrate, the substrate temperature was continuously monitored at the substrate holder/heater by means of a thermocouple and found to be maintained at room temperature during the deposition process. The paper substrates do not show any signs of damage after the deposition of the ZnO thin film. Therefore, choosing the right oxygen working pressure in PED allows us to obtain a modulation of the charge-carrier concentration across the obstacle shadow, which is suitable for a ZnO source−channel−drain structure of a thin film transistor, hence the idea of a reduction in the scale of the experiment described in Figure 1b and already used by us for In_2_O_3_ thin film transistors [9]. A cellulose paper substrate was used since it can also play the role of the gate insulator, as we have shown in [30], and in combination with ZnO thin films, can promise to obtain fully environment-friendly and recyclable devices. Scanning electron microscopy (SEM) images of the paper substrate before and after the ZnO witness (“model”) thin film deposition were acquired with a Tescan Vega XMU-II instrument.

## 3. Results and Discussion

**Figure 2:**
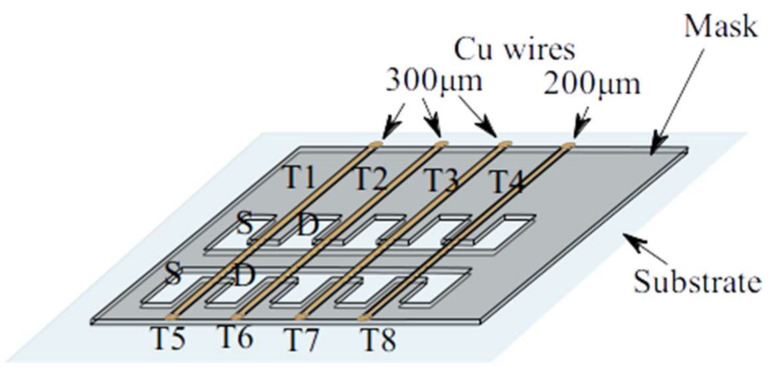
Schematic view of the transistor’s deposition mask with wires on the paper substrate.

In the paper transistor fabricated by PED, the ZnO was used both as an n-type channel semiconductor as well as source and drain electrodes obtained in a single thin film deposition step and gate electrode, respectively. In order to obtain the ZnO source−channel−drain structures, a miniature metal mask was used, placed at a distance of 100 µm from the substrate, where the length of the channel is determined by the thickness of the metal wire behind which the self-assembled deposition is made (Figure 1b). The mask was designed to obtain eight transistors in a single deposition step, arranged in two rows, with four wires, thus causing the formation of eighth channels. Three wires have a thickness of 300 µm, one of 200 µm corresponding to the transistors numbered T4, respectively, T8 in Figure 2, and their diameters directly determine the channel length, while the channel width is determined by the metallic mask itself. For space economy, the drain contact for one transistor is also the source contact for the next transistor in the same row near it.

We have carried out studies to characterize the composition, doping, surface morphology, crystallinity, and optical and electrical properties of the undoped and doped ZnO thin films grown by PED [5,20,29], showing that the electrical and optical properties of films are tunable as a function of the growth parameters. Summarizing our previous work on the large obstacle “model” self-assembled structure [29], Rutherford backscattering spectrometry (RBS) was employed for the determination of the composition and thickness of transparent ZnO thin films as a function of the position on the substrate in the obstacle shadow and in the non-shadowed regions. The composition was Zn_1_O_1_ within the limit of oxygen precision (4%), without impurities, and the thickness profile indicated a bell shape with a minimum thickness in the obstacle shadow and a maximum at the end of the non-shadowed regions. The deposition rate of the ablated species is reduced behind the mechanical obstacle, resulting in oxygen enrichment compared to the regions outside the obstacle shadow, thus explaining the increased resistivity in the obstacle shadow. Also, the transport properties measured by the Hall effect revealed a resistivity of 6 × 10^−3^ Ω·cm, carrier mobility of 21 cm^2^/Vs, carrier density of 5 × 10^19^ cm^−3^ outside the obstacle shadow, and resistivity of 0.225 Ω·cm, carrier density of 9.9 × 10^17^ cm^−3^, and mobility of 28 cm^2^/Vs in the obstacle shadow, respectively. The optical measurements revealed a transmittance of 90–98% for the obstacle shadow region (the “channel”) and 90–95% for adjacent regions (the “electrodes”) in a spectral range of 300–3000 nm, very suitable for transparent devices. In the same work, we have shown that the band gap determined from the Tauc plot is 3.27 eV behind the obstacle and 3.29 eV in the adjacent regions.

**Figure 3:**
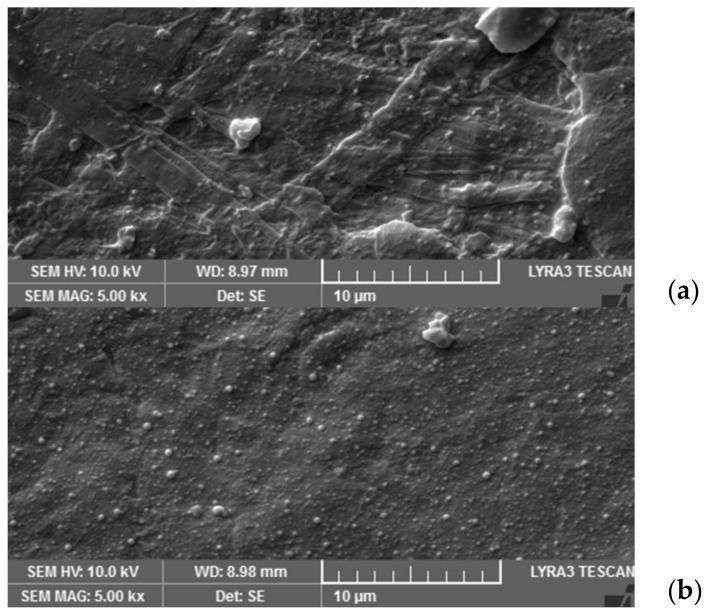
SEM images of the paper substrate before (**a**) and after deposition of the ZnO thin film (**b**).

SEM images of the paper substrate before and after deposition of the ZnO thin film are shown in Figure 3a,b. The image in Figure 3a shows the non-uniform surface of the paper, evidencing disordered fibers and surface, while in Figure 3b, the surface of the paper is continuously covered with the ZnO film. The presence of nano-particulates on the film’s surface is due to the fact that we used an energy of the pulsed electron beam higher than that corresponding to smoother films in order to assure the maximum coverage of the paper’s non-uniform surface. As it has been found in PLD [37,38,39], the presence of a mechanical obstacle in the ablation plasma hinders the ballistic movement of the droplets/nanoparticles from the target to the substrate, resulting in a smooth film area under the obstacle. It is, therefore, expected that the droplet density will decrease drastically in the shadow of the obstacle in a similar way that we have already observed for indium oxide thin films grown under obstacles [9,30].

**Figure 4:**
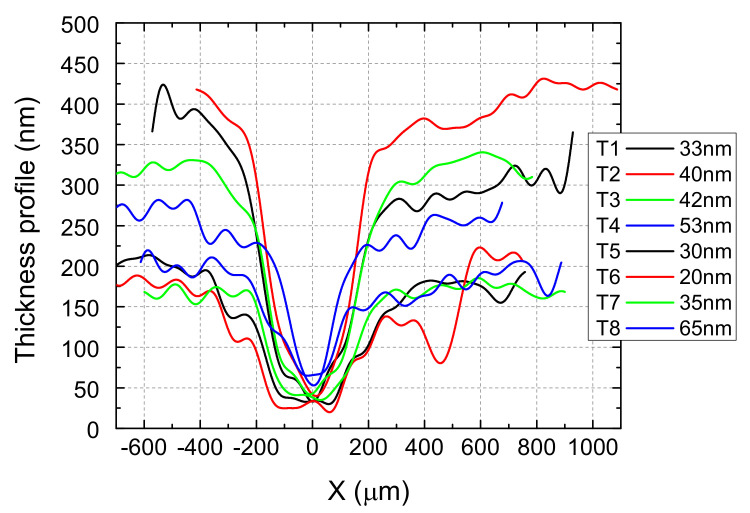
Thickness profiles of self-assembled ZnO channel−source−drain structures; the legend shows the minimum thickness of each channel.

As a preliminary characterization of the ZnO source−channel−drain structures, the channel thicknesses and widths were analyzed by means of the profilometry technique. The thickness profiles of the structures thus formed using metal wires as obstacles are shown in Figure 4, and the minimum channel thickness is between 20 and 65 nm for the eight transistors fabricated in a single deposition. This variation is due to the fact that the surface of the paper has a natural roughness given by the cellulose fibers that compose it, which ensures its flexibility and mechanical and robust dielectric properties [1,2]. The paper substrate for the deposition of the ZnO source−channel−drain structures has not been treated or smoothed before film deposition, as in the references [22,23], or nanopaper was not used [24], as shown in Figure 3a. Moreover, it is also due to the limited uniformity of the deposition on large surfaces, specific to the ablation methods that are anisotropic. Oxide semiconductors exhibit notable stability in contact with cellulose [3,22,23,30], as can also be noted in this case of ZnO thin films grown by PED on a paper substrate. A remarkable coverage of the fibers of the paper with the ZnO thin film deposited at room temperature allows both the necessary transistor components and their suitable functionality to be achieved. This can be explained by the specific characteristic of the PED method in comparison to the magnetron DC or RF magnetron sputtering [6,7,36], pulsed laser deposition [4,8,21], or chemical methods [18,19] to grow smooth, dense, congruent ZnO thin films at low temperatures with tailorable physical properties through the fine control of the deposition parameters [5,20,35].

**Figure 5:**
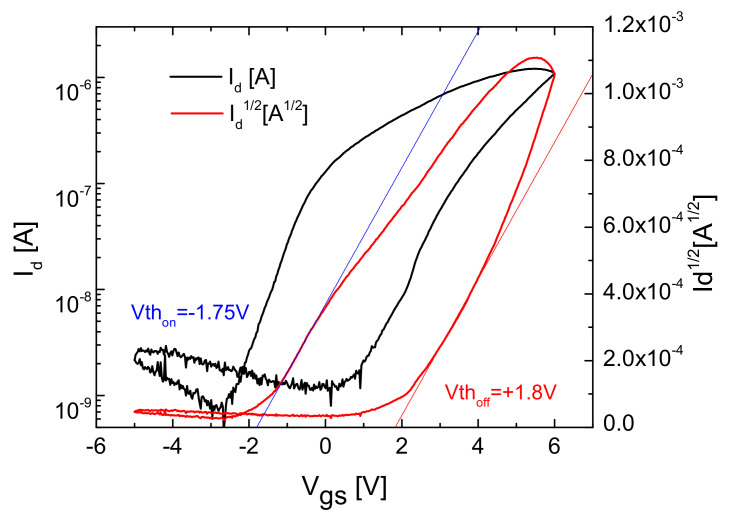
Transfer characteristics (I_d_(V_gs_) and √I_d_(V_gs_)) of a self-assembled field-effect transistor made with ZnO thin films, using paper as both substrate and gate insulator. The black line corresponds to the logarithmic characteristic, and the red line is the square root characteristic used to determine the two threshold values for the “on” state (blue) and “off” state (red).

**Figure 6:**
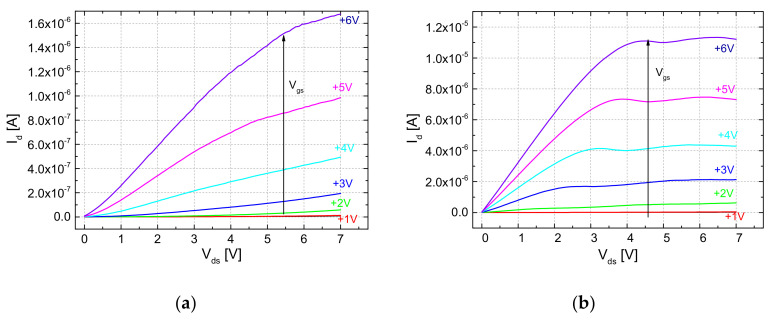
Output characteristics families I_d_(V_ds_) of a ZnO field-effect transistor on paper at different gate voltages: (**a**) in “off” state; (**b**) in “on” state.

With the same deposition mask, self-assembled ZnO thin film field-effect transistors were fabricated by PED at room temperature, using paper as both the substrate and gate insulator. The electrical characteristics of such a transistor are shown in Figure 5 and Figure 6. The measurements are made in the air, in the dark, and at room temperature with two Keithley 2611A SourceMeter systems, a homemade microprobe station using Süss/Cascade Microtec PH-100 magnetic holder micrometric probes, and a LabView acquisition program developed by our team. From the transfer characteristics (Figure 5), it can be noticed that this type of ZnO paper transistor exhibits a memory effect, with two distinct states, “on” and “off”, presenting two different threshold voltages, V_th on_ and V_th off_, similar to the paper-based transistors previously realized by Martins et al. [23], Lim et al. [27,28], and our group [30] for different metal oxide thin films. The maximum drain current is ~11 μA in the “on” state, which allows for obtaining a ratio of I_on_/I_off_ = 970 (and 1370 in the “off” state, respectively).

We define the “off” state, the regime of the memory TFT where the threshold voltage is positive, corresponding to a depleted regime, while in the “on” state, the threshold voltage is negative (enhanced regime).

Previous works related to paper transistors [7,27] suggested that significant carrier scattering by trapped charges at the interface between ZnO and the uneven paper surface could explain the relatively high subthreshold swing. On the other hand, the fiber structure of the cellulose paper could lead to low values of the “on” current in the transistor and I_on_/I_off_ [27,28].

As for the origin of the two states in paper transistors, Martins et al., in [23,40,41,42,43,44,45], identify a charge accumulation in the profound fibers of the cellulose paper that makes the value of the gate capacitance per unit area (C/A) larger than the statically measured C/A. This mechanism was considered similar to that of a supercapacitor, involving an electrical double layer without necessarily relying on an ion circulation like in solid electrolytes [46,47]. For the transient ion-dynamic capacitance in electrolyte-gated transistors, the hysteresis curve goes counter-clockwise, like in [46]: as the gate voltage increases, above-the-threshold-voltage charges are accumulated in the double layer, and the gate capacitance increases due to the decreases in the double-layer thickness. But this lowers the threshold voltage, and when the drain current decreases, the transistor is in enhanced mode as long as the double layer is not depleted by going below the threshold voltage; thus, the hysteresis curve goes counter-clockwise. This also makes the “off” drain current higher in the subthreshold regime in the depleted mode (the “off” state defined above), as seen in Figure 5. The paper transistors, like these presented in this article, have a memory effect with a duration of many days, even weeks, as we showed in [30].

In the literature, the charge-trap/de-trap processes in ZnO charge-trap layers in combination with the IGZO channel layer were also proposed for the development of charge-trap-type non-volatile memory thin film transistors [48,49,50,51]. In our case, even if a charge-trap layer must exist, because the conditions of an irregular channel−insulator interface are present [7,27,28], the charge-trap memory transistors [48,49,50,51] have a shorter duration memory effect and clockwise hysteresis curve. Indeed, while the gate voltage increases, charges are trapped from the channel into the trap layer until it fills, generating a faster increase in the drain current combined with an increase in the threshold voltage, and they are de-trapped when the gate voltage decreases (steep decrease), changing back the threshold voltage. As described for the charge-trapping memory transistors [48,49,51], when *V*_gs_ goes from negative to positive, accumulation of carriers (electrons) in the active channel occurs, followed by tunneling and trapping in the charge-trap semiconductor layer (CTL). When *V*_gs_ goes from positive to negative, the de-trapping of carriers occurs in the CTL, and a depletion layer is created in the channel, so the hysteresis is clockwise, which does not apply to the paper transistor. A shift between the two states occurs, the memory window being the difference between the two *V*_on_.

The drain current in the saturation regime is described by Equation (1) [9,20,21]:

(1) $$I_{d}\left(V_{gs}\right)=\frac{\mu_{\mathrm{nsat}}C_{g}}{2}\frac{W}{L}{\left(V_{\mathrm{gs}}-V_{\mathrm{th}}\right)}^{2}\left(1+\lambda \left(V_{\mathrm{ds}}-V_{\mathrm{dssat}}\right)\right)$$

where *µ*_nsat_ is the charge-carrier mobility at saturation in the channel, *C*_g_ is the capacitance per unit area of the gate insulator, *W* and *L* are the channel’s width and length [34], and λ is a parameter related to the channel length, which can be neglected for the short channel associated to the drain-induced barrier lowering [52,53], which can be neglected in our case.

Assuming λ = 0, a dual representation of *I*_d_(*V*_gs_)—the logarithmic (black line) and square root (red line)—is shown in Figure 5. From the logarithmic representation, we can determine the slope of the curve as a logarithmic sensitivity below the threshold voltage (subthreshold swing in volts/decade) [34], and in square root representation, considering Equation (1), the value of the slope of *I_d_*^1/2^ at *I_d_* = 0 gives the threshold voltage. The transfer characteristics in Figure 5 are measured at *V*_ds_ = 8 V.

For the “on” state, we determined a threshold voltage *V*_th on_ = −1.75 V and a subthreshold swing of S = 1.1 V/decade. In the “off” state, we have *V*_th off_ = +1.8 V and S = 1.34 V/decade, and the carrier mobility at channel saturation, also deduced from Equation (1) using the measured drain current at saturation, is ~25 cm^2^/Vs in both states. This value of the carrier mobility is comparable with the values reported in the literature for ZnO transistors [5,7] and with the ones determined by the Hall effect measurements for ZnO thin films in our previous experiments [29].

Experimenting with mask wire diameters was necessary, as the channel full-width at half-maximum (FWHM) is influenced by the oxide target type due to the scattering of the plasma species in the background gas (influenced by the atomic mass). As the atomic mass of Zn is two times lighter than In ions, we expected that the channel FWHM should have been smaller than the wire diameter, more than in the case of In_2_O_3_ transistors [9]. We obtained 200 µm channels under the 200 µm wire, but 400 µm FWHM channels under the 300 µm wires. As can be seen in Figure 2 (mask schematic) and Figure 4 (profilometry), the only two transistors with a 200 µm wire mask are T4 and T8 (200 µm FWHM channel). As an ablation deposition method, the PED uniformity of the deposited film is limited due to the anisotropy of the method, so the T1−T4 line has a film thicknesses outside the wire shadow (source−drain thickness) of 250–400 nm, while the film thickness for the T5–T8 line is in the range of 150–200 nm (Figure 4). The film is grown in oxygen, and the oblique incidence far from the plasma plume axis leads to smaller thicknesses and also to a slight enrichment in oxygen, more important for small thicknesses, as explained for the indium oxide transistors [9]. This explains the fact that the T8 channel has both smaller conductivity and the smallest length, leading to better performances, so we featured it as the most representative.

In Figure 6a,b, the output characteristics of the ZnO self-assembled field-effect transistor using paper as both substrate and gate insulator are measured in strictly the same conditions: the same range of the drain voltage and the same values of the gate voltage. One can observe that the maximum value of the drain current in the “on” state is ten times greater, the drain current slope is higher, and the output drain current saturation occurs at less than 3 V drain-source, compared to the “off” state where there is no drain current saturation.

We propose for applications, the following “on/off” state interrogation method: the application of a gate voltage between V_th on_ and V_th off_, which we call “interrogation voltage”. If the drain current is smaller than 10 nA, the memory TFT will be in the “off” state, while if it is greater than 100 nA, it is in the “on” state. Applying a gate voltage of -V_ds_ switches the transistor to an “off” state, while V_gs_ = V_ds_ switches the transistor to an “on” state.

The hysteresis behavior of transfer characteristics was explained by the charge-trapping electrons/holes model inside the bulk paper and at the interface between the channel and gate dielectric, enhanced when a low-temperature fabrication is used [3,7,23,27,28,30]. As a matter of fact, many studies have been carried out to understand the mechanism behind the memory effect in transistors made with and on paper [40,41,42,43,44,45,46,47,48,49,50,51,52,53,54,55]. It has been found that the main mechanism responsible for the hysteresis effect, i.e., the increase of S and the hysteresis width (ΔV), is given by the spatial drift of the space charge within the paper bulk. The large drain current of the paper transistors can also be explained by the enhancement of the capacitance per unit area of the paper due to its discrete dielectric structure, constituted by interconnected coated and uncoated fibers that form a multilayer structure, as stated by R. Martins et al. [23,40,41,42,43,44], similar to a supercapacitor. More than that, this fiber-structured supercapacitor will enhance the hysteresis by a large accumulation of charge, resulting in the observed “memory effect” [45]. The type of paper used for the transistors has been investigated in detail, highlighting the role of the fiber structure, fiber dimensions, compactness, porosity, and surface finish [56,57,58]. We also note that the “on”−”off” commutation state of ZnO thin film transistors is retained for several days, even in the absence of electrical supply, similar to the In_2_O_3_ paper transistors [30], which opens the possibility of use in passive memory circuits [23,40,41,42,43,44,45,46,47,48,49,50,51,52,53,54,55]. 

Other mechanisms related to the hysteresis and memory effect are also presented in the literature [46,47,48,49,50,51,52,53,54,55]. In addition, charge-trap/de-trap processes in ZnO charge-trap layers have been proposed to explain the mechanisms behind the non-volatile memory thin film transistors [48,49,50,51]. In particular, when the direction of the hysteresis is counter-clockwise, the mechanisms are associated with mobile ions, as discussed in [46,47]. Such electrolyte-gated transistors are more and more used as switching elements, artificial synapses, and memristive systems [46].

J. Jiang et al. [59] used a similar approach of a shadow mask for the self-alignment fabrication of an indium tin oxide (ITO) transistor on paper. They obtained an “in-plane” ITO gate transistor where the 200 nm ITO source, drain, and gate terminals were placed on the same plane by using a metallic shadow mask during a one-step deposition at room temperature by magnetron sputtering. The ITO transistor channel was self-assembled between the ITO source and drain electrodes, i.e., grown in the shadow of the metallic shadow mask during deposition, and placed at 50 µm above the paper substrate. A distance of 300 µm was used between the gate and source electrodes, while the channel had a length of 50 µm and a width of 1000 µm, respectively. This method differs from our method by the fact that in their case, the paper was used only as a substrate, the gate dielectric being a 1.5 µm thick SiO_2_ layer grown by plasma-enhanced chemical-vapor deposition on a silver gate electrode. These two layers avoided the problems related to non-uniform paper surfaces since the quality of the interface between the channel and gate dielectric could influence the performance of transistors [59,60], a similar approach being used for other transistors [61,62,63]. Therefore, the ITO “in-plane” TFTs had a high field mobility of 22.4 cm^2^/V s, comparable to that obtained in this work for ZnO TFT but a large on/off ratio of 8 × 10^5^, a subthreshold swing of 192 mV/decade, and a 2.0 V operating voltage, respectively [59].

When amorphous IGZO thin film transistors were fabricated on paper by magnetron sputtering at room temperature [27], following the pioneering work [7] with paper used as both a gate dielectric and a substrate, the photolithography and lift-off processes were used to pattern the source and drain electrodes on the IGZO channel layer. These wet processes have led to chemical absorption in the paper during electrode patterning, accentuating the unevenness of the paper surface and thus increasing the resistance between the source and drain contacts. Despite these problems, the TFT performance, also related to the IGZO channel material, has shown a saturation mobility of 35 cm^2^/V, a threshold voltage of 3.75 V, a subthreshold gate-voltage swing of 2.4 V/decade, and a drain current on-to-off ratio of 10^4^, respectively.

Following this work, Lim et al. [28] spin-coated a water and solvent barrier onto the paper substrate, improving the surface roughness, and used a SiOx as a gate dielectric, not the paper itself. The threshold voltage (~1.9 V) and gate-voltage swing (0.65 V/decade) were lowered as a result of a smoother paper surface and, hence, of the films deposited on it, reducing the interface trap density between the channel and gate dielectric. The IGZO channel saturation mobility had a value of 1.2 cm^2^/V s, lower than that obtained when paper was used as both a gate dielectric and a substrate, but this comparison does not take into account the different sizes of transistors and thicknesses, which also influences their performance.

Room-temperature junctionless thin film transistors on paper substrate gated with chitosan electrolyte have been fabricated and presented a 1.0 V operating voltage [64]. This was explained by a high gate-specific capacitance value related to the electrical double-layer effect induced by mobile ions. When beeswax was used as the gate dielectric of indium-zinc-oxide thin film homojunction transistors on paper substrates, the counter-clockwise hysteresis window was attributed to mobile protons in the beeswax layer [65]. Poly-Si thin film transistors were successfully printed at 100 °C as both a p-channel and n-channel on paper with field-effect mobilities of 6.2 and 2.0 cm^2^/V s, respectively [66], and the poly-Si was patterned using an excimer laser based on additive selective crystallization of the precursor film. A review of electronics on paper highlighted the advances in the manufacturing of electronic components on paper for various applications and the challenges related to the roughness and absorptive surface of paper [67].

These results strengthen the fact that our approach of making self-assembled ZnO structures for thin film transistors with and on paper at room temperature in a single deposition improves some of the problems raised by transistor fabrication. Such ZnO thin film transistors are also ideal as components for single-use smart labels with low energy consumption and single-use electronics, such as flexible, disposable smart labels and sensors for portable devices.

Refinements in the composition, density, and functionalization of the cellulose-based paper will reduce the operating voltage and leakage current, improving the performance of inorganic or organic transistors. Reducing the film’s thickness, adding a surface passivation layer, and modulating the conductivity of the film are possible strategies for suppressing the hysteresis effect in paper transistors. The following approaches can also be used: depositing charge-trap/de-trap films in front of the channel [48], geometric effects of the ZnO acting as a charge-trap layer [51], optimizing the deposition and fabrication process [54], and planarizing the paper surface [55]. Another possibility would be to use hysteresis for neuromorphic applications, and further development of artificial synaptic thin film transistors using transparent zinc oxide [68,69] will lead to integrating electronic devices with paper [70].

## 4. Conclusions

In this study, functional transparent self-assembled ZnO structures for thin film transistors on paper were fabricated by PED in a single deposition step at room temperature. ZnO has been used as both an n-type channel semiconductor and source, drain, and gate electrode in papered thin film transistors, minimizing steps in the thin film transistor manufacturing process and reducing the cost. Electrical measurements were performed on the self-assembled transistor structures made using both paper as the substrate and gate dielectric. Following these measurements, the deposition parameters in PED were optimized in terms of the electrical properties of the self-assembled structures. Transistors with these self-assembled ZnO thin film structures on paper have been demonstrated, and their functionality has been proven; like in the case of In_2_O_3_ paper transistors, these transistors exhibit a “memory effect” [30], similar to other transistors with and on paper reported in the literature but using thin films of different metal oxides [22,28]. Given the irregular-by-nature surface of the paper without preliminary treatment and the anisotropy of the ablation plasma in the PED thin film deposition, the channel geometry reproducibility, as seen in Figure 3, is satisfactory.

As discussed above, the memory effect can be explained by various mechanisms like the supercapacitor charge-trap/de-trap process, but the most likely is due to movable ions, as evidenced by the counter-clockwise hysteresis of the transfer characteristics. [46]. However, a much smaller hysteresis due to charge-trapping by the irregular cellulose fibers could also be present, a fact proven by the high threshold voltages and the small I_on_/I_off_ and “on” currents. Further improvements in device characteristics could be made by depositing charge-trap/de-trap films before the channel, the study of areal geometric effects of a ZnO acting as a charge-trap layer [51], optimization of the deposition and fabrication process [54], and planarization of the paper surface by using organic/inorganic hybrid layers [55].

The determined off–on switching gate voltage is +6 V at V_ds_ = +8 V, and the off–on gate voltage is −2 V. A 1.6 μA maximum drain current was determined in the “off” state, and 11.5 μA in the “on” state of the thin film ZnO transistor on and with paper. The saturation charge-carrier mobility is 25 cm^2^/Vs in both states, consistent with the Hall effect measurements. The safe “interrogation” gate voltages for such a transistor used as a memory device are in the range between V_th on_ = −1.75 V and V_th off_ = +1.8 V, which would yield a drain current of 0.05–0.1 μA in the “on” state and 1–10 nA in the “off” state for V_ds_ = 8 V, as determined from the transfer characteristics. In the case when the transistor is used as an amplifying device, the subthreshold swing has values between 1.1 and 1.34 V/decade.

We are considering some improvements, like shortening the channel (by decreasing the wires’ diameters) with the purpose of reducing the working voltage, which will leave the possibility to also decrease the width of the channel and improve the overall performance of the transistors. This will require optimizations, as the memory effect relies specifically on the channel to be deposited directly on paper, and the fiber dimensions impose geometrical limits in this reduction. We are also planning to reduce the surface of the shadow mask in order to minimize the impact of the anisotropy of the PED method for multi-transistor devices. Another approach using magnetron RF-sputtering for large surface deposition is under work.

Cunha et al. [3] demonstrated a non-toxic and low-cost process for the recycling of devices based on papered thin film transistors. Cellulosic materials were disintegrated relatively easily in water to form cellulose, which can then be recycled and reused to produce new paper substrates for electronic devices. Although the recycled paper has a slightly lower capacitance and a higher hysteresis than the original, a paper transistor can still operate satisfactorily. Such an approach could be envisaged for the ZnO paper transistors fabricated by PED, considering that cellulose is 100% biodegradable and zinc oxide is also recyclable. Even if the current capability of ZnO paper transistors subject to this research is almost two orders of magnitude smaller than for our In_2_O_3_ paper transistors [30], they are perfectly usable in applications like disposable smart labels and other very low-power disposable electronics like flexible displays and sensors for wearable devices, in trend with the current “green” paper-based electronics.

## Data Availability

The data presented in this study are available from the corresponding author upon reasonable request.
